# Selection and Validation of Reference Genes For qRT-PCR Analysis of *Rhopalosiphum padi* (Hemiptera: Aphididae)

**DOI:** 10.3389/fphys.2021.663338

**Published:** 2021-04-14

**Authors:** Mengyi Li, Xinan Li, Chao Wang, Qiuchi Li, Saige Zhu, Yunhui Zhang, Xiangrui Li, Fengshan Yang, Xun Zhu

**Affiliations:** ^1^Heilongjiang Provincial Key Laboratory of Ecological Restoration and Resource Utilization for Cold Region, School of Life Sciences, Heilongjiang University, Harbin, China; ^2^Institute of Plant Protection, Chinese Academy of Agricultural Sciences, Beijing, China; ^3^State Key Laboratory for Biology of Plant Diseases and Insect Pests, Beijing, China; ^4^School of Resource and Environmental Sciences, Henan Institute of Science and Technology, Xinxiang, China

**Keywords:** *Rhopalosiphum padi*, qRT-PCR, reference gene, RefFinder, normalization

## Abstract

*Rhopalosiphum padi* (L.) (Hemiptera: Aphididae) is an important cosmopolitan pest in cereal crops. Reference genes can significantly affect qRT-PCR results. Therefore, selecting appropriate reference genes is a key prerequisite for qRT-PCR analyses. This study was conducted to identify suitable qRT-PCR reference genes in *R. padi*. We systematically analyzed the expression profiles of 11 commonly used reference genes. The ΔCt method, the BestKeeper, NormFinder, geNorm algorithms, and the RefFinder online tool were used to evaluate the suitability of these genes under diverse experimental conditions. The data indicated that the most appropriate sets of reference genes were *β-actin* and *GAPDH* (for developmental stages), *AK* and *TATA* (for populations), *RPS18* and *RPL13* (for tissues), *TATA* and *GAPDH* (for wing dimorphism), *EF-1α* and *RPS6* (for antibiotic treatments), *GAPDH* and *β-actin* (for insecticide treatments), *GAPDH*, *TATA*, *RPS18* (for starvation-induced stress), *TATA*, *RPS6*, and *AK* (for temperatures), and *TATA* and *GAPDH* (for all conditions). Our study findings, which revealed the reference genes suitable for various experimental conditions, will facilitate the standardization of qRT-PCR programs, while also improving the accuracy of qRT-PCR analyses, with implications for future research on *R. padi* gene functions.

## Introduction

The bird cherry-oat aphid, *Rhopalosiphum padi* (L.) (Hemiptera: Aphididae), is one of the most important wheat pests (Leather et al., 1989). It can decrease crop yield directly by feeding on plants and indirectly by transmitting the barley yellow dwarf virus (Smyrnioudis et al., 2007). Additionally, *R. padi* adapted to the long-term use of insecticides and developed insecticide resistance, making it difficult to control (Zuo et al., 2016; Zhang et al., 2017; Gong et al., 2020; Umina et al., 2020). The need for environmentally friendly pest control methods to ensure the sustainable production of agriculturally and economically important crops has compelled researchers to investigate insect pests at the ecological, physiological, and molecular levels over the past few decades. This research has resulted in important advances in several areas, including transcriptomics (Duan et al., 2017; Nazar et al., 2020; Wang R. et al., 2020), proteomics (Vandermoten et al., 2014; Prajapati et al., 2020), insecticide resistance (Zhang et al., 2016; Gong et al., 2020), insect–endosymbiont interactions (De Moraes et al., 2018; Liu et al., 2019), RNA interference (Kola et al., 2019; Ma et al., 2020), and gene functions (Chen A. et al., 2020; Pan et al., 2020). Molecular analyses of *R. padi* have revealed many important genes (Chen and Han, 2006; Zhang et al., 2016; Fan et al., 2017; Balakrishnan et al., 2018). However, additional research is required to thoroughly clarify the mechanisms regulating the expression of these genes, which may provide insights into the molecular basis of *R. padi* insecticide resistance and enable the development of improved pest control strategies.

Quantitative real-time PCR (qRT-PCR) is the best choice for analyzing gene expression and the transcriptome because of its sensitivity, reproducibility, and specificity. Moreover, it can be conducted on high-throughput platforms (Koramutla et al., 2016; Li et al., 2016). Reliable qRT-PCR results are based on an accurate transcript normalization (Han et al., 2013). Many factors considerably influence the threshold cycle (Ct) values, including RNA quality and quantity, variable transcriptional efficiencies, primer characteristics, and PCR conditions (Udvardi et al., 2008; Bustin et al., 2009). Consequently, identifying appropriate and reliable reference genes to serve as internal controls is essential for normalizing expression levels (Bustin, 2002; Pinheiro and Siegfried, 2020). In most analytical methods, the use of reference genes can eliminate the differences in sample purity and concentration, thereby enabling comparisons of target gene expression between samples (Radonić et al., 2004).

Several housekeeping genes involved in basic, ubiquitous cellular functions have been used to normalize gene expression (Eisenberg and Levanon, 2013; Adeyinka et al., 2019), including genes encoding elongation factor 1α, β-actin, glyceraldehyde-3-phosphate dehydrogenase, glutathione S-transferase, ribosomal proteins, and β-tubulin. Ideal reference gene expression levels are stable and unaffected by changes to experimental and environmental conditions (Bustin, 2000; Hong et al., 2008). If the suitability of these genes under specific experimental conditions is not carefully considered, the resulting qRT-PCR data may be inaccurate or inconsistent (Kang et al., 2017). There is no reference gene that is appropriate for all gene expression analyses (Kubista et al., 2006). Consequently, the potential utility of reference genes must be systematically evaluated under specific conditions. Several methods and programs have been developed to evaluate the stability of reference genes, including the ΔCt method (Silver et al., 2006), BestKeeper (Pfaffl et al., 2004), NormFinder (Andersen et al., 2004), geNorm (Vandesompele et al., 2002b), and RefFinder, which is a web-based tool (Xie et al., 2012).

Considering the importance of reference genes for qRT-PCR, previous studies validated reference gene sets in various insect species, including *Lymantria dispar* (Yin et al., 2020), *Drosophila melanogaster* (Kim et al., 2020), *Pagiophloeus tsushimanus* (Chen C. et al., 2020), *Phenacoccus solenopsis* (Zheng et al., 2019), *Chilo partellus* (Adeyinka et al., 2019), *Harmonia axyridis* (Yang et al., 2018), *Henosepilachna vigintioctomaculata* (Lu et al., 2018), *Liriomyza trifolii* (Chang et al., 2017), *Myzus persicae* (Kang et al., 2017), *Bradysia odoriphaga* (Shi et al., 2016), *Lipaphis erysimi* (Koramutla et al., 2016), *Helicoverpa armigera* (Shakeel et al., 2015), *Sesamia inferens* (Lu et al., 2015), and *Spodoptera exigua* (Zhu et al., 2014). In a previous study, the gene expression stability of four potential housekeeping genes was evaluated for viruliferous winged and wingless *R. padi* adults (Wu et al., 2014), which is an insufficient number of genes.

In this study, the following 11 commonly used reference genes were analyzed to assess their suitability for normalizing qRT-PCR data for *R. padi*: *elongation factor 1α* (*EF-1α*), *beta* actin (*β-actin*), *arginine kinase* (*AK*), *TATA-box binding protein* (*TATA*), *glyceraldehyde-3-phosphate dehydrogenase* (*GAPDH*), *glutathione S-transferase* (*GST*), *ribosomal protein L13* (*RPL13*), *ribosomal protein S6* (*RPS6*), *ribosomal protein S18* (*RPS18*), *18S ribosomal RNA* (*18S*), and *28S ribosomal RNA* (*28S*). Additionally, the effects of the following factors on reference gene expression were evaluated: developmental stage, population, tissue, wing dimorphism, antibiotic treatment, insecticide treatment, temperature, and starvation. The objective of this study was to identify different sets of suitable reference genes for *R. padi* gene expression analyses under various experimental conditions. Our results may be useful for developing a more rigorous approach to normalizing *R. padi* qRT-PCR data.

## Materials and Methods

### Insects

The *R. padi* clones included in this study were originally collected from Shizuishan (Ningxia), China (39°01′58.81″N, 106°37′75.60″E) in 2018. The clones were reared on Lunxuan 987 wheat seedlings in a thermostatic chamber maintained at 20 ± 2°C and 60% relative humidity, with a 16-h light: 8-h dark photoperiod.

### Analyzed Factors

#### Developmental Stage

Three replicates of wingless *R. padi* were collected at the following stages: 60 first instar nymphs, 60 second instar nymphs, 60 third instar nymphs, 40 fourth instar nymphs, and 40 adults. The samples were flash frozen in liquid nitrogen and stored at −80°C until analyzed by qRT-PCR.

#### Population

Insects collected in Shizuishan (Ningxia, 39°01′58.81″N, 106°37′75.60″E) and Langfang (Hebei, 39°8′9.8″N, 116°10′4.05″E), China in 2018 were examined to assess the effects of geography on gene expression. These two locations are separated by more than 1,000 km. For each population, three replicates of 30 wingless adults were collected, flash frozen in liquid nitrogen, and stored at −80°C until analyzed by qRT-PCR.

#### Tissue

A dissection needle, tweezers, and a stereo microscope were used to collect the head, thorax, and abdomen from wingless *R. padi* adults. For each body part, three replicates of 100 tissue samples were collected, flash frozen in liquid nitrogen, and stored at −80°C until analyzed by qRT-PCR.

#### Wing Dimorphism

Three replicates of 40 winged and wingless *R. padi* adults were collected, flash frozen in liquid nitrogen, and stored at −80°C until analyzed by qRT-PCR.

#### Antibiotic Treatment

Wingless *R. padi* adults were fed a 30% sucrose solution containing 50 μg/mL rifampicin or an antibiotic-free sucrose solution (control) (25 aphids per feeder) for 48 h (Wilkinson and Ishikawa, 2001). For the control and treatment groups, three replicates of 40 adults were collected, flash frozen in liquid nitrogen, and stored at −80°C until analyzed by qRT-PCR.

#### Insecticide Treatment

Wingless *R. padi* adults were treated with one of the following three insecticides: beta-cypermethrin (774.57 mg/L), imidacloprid (14.28 mg/L), and sulfoxaflor (4.79 mg/L). These concentrations were used because a bioassay indicated they are lethal to 30% of the population. The 1% insecticide stock solutions prepared in acetone were serially diluted with water (containing 0.1% Tween-80) to produce five concentrations. Water (containing 0.1% Tween-80) was used as the control solution. Wheat leaves with wingless *R. padi* adults were immersed in the prepared solutions for 3–5 s and then placed on moistened filter paper in a Petri dish (9 cm diameter). For each concentration, the mortality rate based on three replicates of 30 aphids was calculated. For the control and treatment groups, three replicates of 40 adults were collected, flash frozen in liquid nitrogen, and stored at −80°C until analyzed by qRT-PCR.

#### Starvation

Wingless *R. padi* adults were placed in Petri dishes (9 cm diameter) with no food for a 32-h incubation in a thermostatic chamber. The control (satiated) group comprised wingless aphids able to feed on wheat seedlings. For the two groups, three replicates of 40 adults were collected, cultured, and stored as described earlier. The mortality rate among the starved aphids was approximately 10%.

#### Temperature

Petri dishes containing wheat leaves with wingless *R. padi* adults were divided into five groups for a 24-h exposure to various temperatures (4, 10, 15, 20, and 30°C). For each temperature, three replicates of 40 adults were collected, cultured, and stored as described earlier. The aphids survived all temperature treatments.

### Total RNA Extraction and cDNA Synthesis

Total RNA was extracted using the TRNzol Universal Reagent as described by the manufacturer (Tiangen, Beijing, China). The ratio of the absorbance at 260 and 280 nm was 1.981–2.121, indicating the extracted RNA was pure. Next, 1 μg RNA was used as the template to synthesize first-strand cDNA with the FastKing gDNA Dispelling RT SuperMix (Tiangen) following the manufacturer-recommended protocol. The synthesized cDNA was stored at −20°C.

### Primer Design and Quantitative Real-Time PCR

A qRT-PCR assay was completed using the Talent qPCR PreMix (SYBR Green; Tiangen) and the CFX Connect Real-Time system (Bio-Rad, Hercules, CA, United States). Information regarding the primers for the *EF-1α*, *18S*, and *28S* genes has been published by NCBI. The primers for the *β-actin* and *GAPDH* genes were designed in a previous study (Wang et al., 2018). The primers for the other target genes were designed based on our unpublished *R. padi* RNA sequencing data. Details regarding the qRT-PCR primers are provided in Table 1. For each sample, the cDNA was prepared as a 60-ng/μL working solution. The qRT-PCR was completed in a 20-μL reaction volume comprising 10 μL 2× Talent qPCR PreMix, 0.6 μL forward primer (100 μM), 0.6 μL reverse primer (100 μM), 0.6 μL cDNA working solution, and 8.2 μL RNase-free ddH_2_0. The PCR program was as follows: 95°C for 3 min; 40 cycles of 95°C for 5 s and 60°C for 15 s. For each primer, standard curves were produced using a five-fold dilution series of cDNA as the template according to the linear regression model. The qRT-PCR analyses were completed with three biological replicates and three technical replicates.

**TABLE 1:** Primer sequences and amplicon characteristics of the 11 reference genes in *R. padi* samples.

Gene symbol	Gene name	Gene ID	Primer sequences (5′→3′)	^a^L (bp)	^b^E (%)	^c^*R*^2^	Slope
*EF-1α*	*Elongation factor 1 alpha*	KY612590	F: CTGTTGCTTTCGTTCC	227	91.64	0.9952	−3.54
			R: GACTGTTCCAATACCTCC				
*β-Actin*	*Beta actin*	KJ612090.1	F: TGAGACATTCAACACCCCTG	132	98.23	0.9975	−3.37
			R: CCTTCATAGATTGGGACAGTG				
*AK*	*Arginine kinase*	XM_026962165.1	F: GGAAGAAGGGTGGTGT	178	100.27	0.9985	−3.32
			R: CAGCGTCAGGAGCATA				
*TATA*	*TATA-Box binding protein*	XM_026955067.1	F: TGTCGGCTTGACCTAA	262	99.23	0.9983	−3.34
			R: ACAACTGCCAACCATG				
*GAPDH*	*Glyceraldehyde-3-phosphate dehydrogenase*	KJ612091.1	F: GCTCCATTAGCCAAGGTTATTC	136	90.6	0.9992	−3.57
			R: CAGCACCTCTACCATCTCTCC				
*GST*	*Glutathione S-transferase*	XM_026965253.2	F: ATGACGGTTATTTTGT	186	98.71	0.9822	−3.35
			R: CAGGTCTTTTTGCTTG				
*RPL13*	*Ribosomal protein L13*	XM_025340592.1	F: CAAAGACTGGCAACG	240	107.77	0.9993	−3.15
			R: CCAATGGTACGAGCA				
*RPS6*	*Ribosomal protein S6*	XM_026959847.1	F: ACTCGGTGATGAATGG	138	104.59	0.9998	−3.22
			R: GGGCGATAACAAGAAT				
*RPS18*	*Ribosomal protein S18*	XM_026959879.1	F: CACATCTTGCGTATCCT	148	98.22	0.9997	-3.37
			R: TACATTCTCCAGCCCTC				
*18S*	*18S Ribosomal RNA*	KJ612093	F: ACGCATCTTTCAAATGTCTG	125	98.88	0.9953	−3.37
			R: TGTGGTAGCCGTTTCTCA				
*28S*	*28S Ribosomal RNA*	AF487719	F: AACAACCGTGATTCCC	101	92.03	0.9994	−3.53
			R: CGCCACAACTCCCATA				

*^a^Amplicon length. ^b^qRT-PCR efficiency determined using the standard curve method. ^c^Regression coefficient calculated using the regression line of the standard curve.*

### Data Analysis

Gene expression levels were calculated as the number of cycles needed for the amplification to reach a fixed threshold in the exponential phase of the PCR (i.e., Ct). The threshold was set to 500 for all genes. The stability of the 11 housekeeping genes was evaluated using the geNorm, NormFinder, and BestKeeper algorithms and the comparative ΔCt method. Finally, we compared and ranked the tested candidate reference genes with the web-based RefFinder analytical tool^1^.

## Results

### Amplification Efficiencies

The qRT-PCR data indicated that all 11 candidate reference genes were expressed in the *R. padi* samples. The PCR products for these genes were visualized as a single amplicon of the expected size for each primer pair on 1% agarose gels. Moreover, our study used five-point standard curves with known RNA concentrations to estimate the amplification efficiencies. Gene-specific amplification was confirmed by a single peak and the lack of primer dimer peaks in the melting-curve analysis. The amplification efficiencies (*E*) of these genes ranged from 90.6 to 107.77%. The regression coefficient (*R*^2^) was greater than 0.9822 (Table 1).

### Expression Profiles of Candidate Reference Genes

**FIGURE 1:**
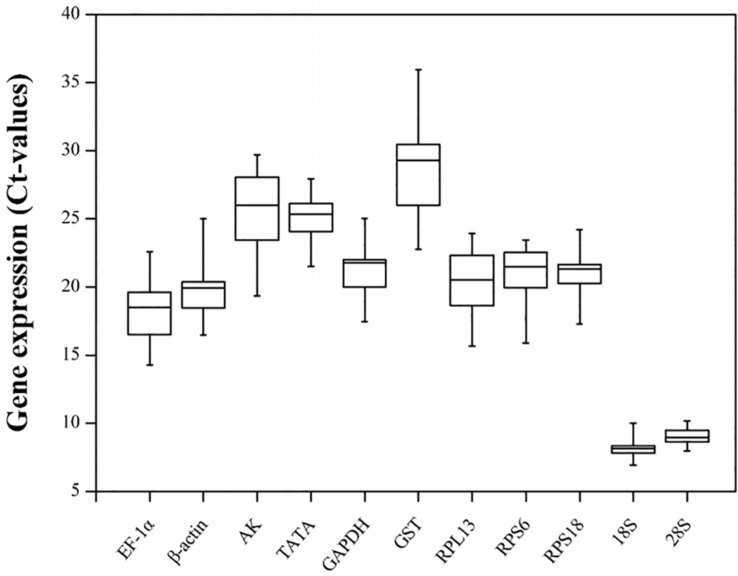
Candidate reference gene expression profiles in *R. padi.* Expression data are presented as mean Ct values for duplicate samples. Whiskers represent the maximum and minimum values. The lower and upper borders of boxes represent the 25th and 75th percentiles, respectively. The line across the box indicates the median Ct value.

The gene expression levels of the 11 candidate reference genes revealed a broad Ct range under all experimental conditions (Figure 1). The Ct values ranged from 6.93 for the *18S* gene to 35.94 for the *GST* gene. The mean Ct values for the *18S* and *28S* genes were 8.08 and 9.08, respectively, which were much lower than the Ct values of the other genes. Both genes were highly expressed in all conditions. The other nine candidate reference genes were expressed at moderate levels. Specifically, the mean Ct values for the *EF-1α*, *β-actin*, *RPL13*, *RPS6*, *RPS18*, *GAPDH*, *TATA*, *AK*, and *GST* genes were 18.29, 19.81, 20.46, 20.85, 20.95, 21.33, 25.16, 25.74, and 28.50, respectively.

### Stability of Candidate Reference Genes

#### Developmental Stage Analysis

**FIGURE 2:**
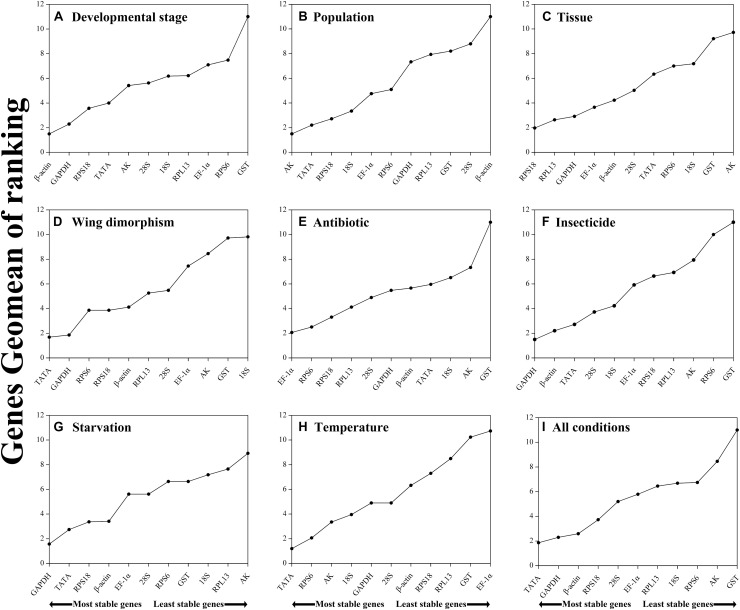
Stability of candidate reference genes in *R. padi* under various experimental conditions. In a RefFinder analysis, increasing Geomean values correspond to decreasing gene expression stability. The Geomean values for the following *R. padi* samples are presented: **(A)** Developmental stage: samples for all developmental stages; **(B)** Population: adult samples from different geographical populations; **(C)** Tissue: samples for different tissues of wingless adults; **(D)** Wing dimorphism: samples for winged and wingless adults; **(E)** Antibiotic treatment: adult samples treated with different antibiotics; **(F)** Insecticide treatment: adult samples treated with different insecticides; **(G)** Starvation: fed and unfed adult samples; **(H)** Temperature: adult samples exposed to different temperatures; **(I)** All conditions: all samples for all treatments. The candidate reference genes are as follows: *EF-1α*, *elongation factor 1α*; *β-actin*; *AK*, *arginine kinase*; *TATA*, *TATA-box binding protein*; *GAPDH*, *glyceraldehyde-3-phosphate dehydrogenase*; *GST*, *glutathione S-transferase*; *RPL13*, *ribosomal protein L13*; *RPS6*, *ribosomal protein S6*; *RPS18*, *ribosomal protein S18*; *18S*, *18S ribosomal RNA*; *28S*, *28S ribosomal RNA*.

**FIGURE 3:**
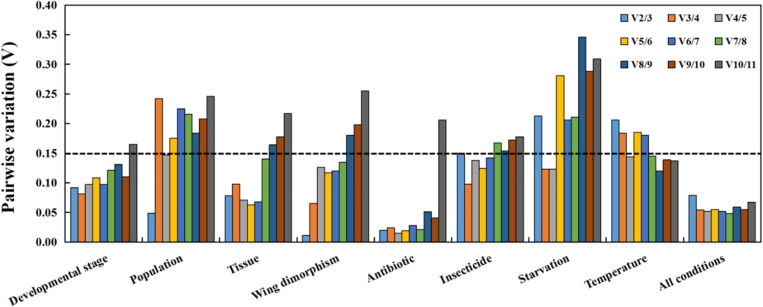
Optimal number of reference genes for accurate normalization as determined by geNorm. The Vn/n+1 value indicates the pairwise variation (*Y*-axis) between two sequential normalization factors and was used to determine the optimal number of reference genes for an accurate data normalization. *A*-value < 0.15 indicates that an additional reference gene will not significantly improve the normalization.

Regarding the analyzed developmental stages, geNorm, NormFinder, and the ΔCt method, but not BestKeeper, indicated that *β-actin* and *GAPDH* were the most stable genes (Table 2). The BestKeeper analysis identified *28S* and *18S* as the most stable genes. In contrast, *GST* was the least stable gene. The RefFinder analysis indicated the rank order for reference gene stability was as follows (most to least stable): *β-actin*, *GAPDH*, *RPS18*, *TATA*, *AK*, *28S*, *18S*, *RPL13*, *EF-1α*, *RPS6*, and *GST* (Figure 2A). The geNorm analysis revealed that all pairwise variation values were less than the proposed 0.15 cut-off, except for V10/11 (Figure 3). A value less than 0.15 indicates that adding another reference gene will not obviously change the normalization. The RefFinder analysis indicated *β-actin* and *GAPDH* are required for normalizing target gene expression levels in different *R. padi* developmental stages (Table 3).

**TABLE 2:** Rank order of the *R. padi* candidate reference genes under various experimental conditions.

Experimental Condition	Rank	ΔCt		BestKeeper		NormFinder		geNorm	
		Gene	SV	Gene	SD	Gene	SV	Gene	SV
Developmental stages	1	*β-actin*	0.92	*28S*	0.45	*β-actin*	0.16	*β-actin/*	0.33
	2	*GAPDH*	1.00	*18S*	0.50	*GAPDH*	0.45	*GAPDH*	
	3	*RPS18*	1.04	*AK*	0.96	*RPS18*	0.59	*RPS18*	0.43
	4	*TATA*	1.08	*TATA*	1.06	*TATA*	0.61	*TATA*	0.48
	5	*RPL13*	1.16	*β-actin*	1.08	*RPL13*	0.69	*EF-1α*	0.59
	6	*AK*	1.22	*GAPDH*	1.29	*AK*	0.78	*RPL13*	0.72
	7	*EF-1α*	1.25	*RPS18*	1.29	*RPS6*	0.90	*RPS6*	0.83
	8	*RPS6*	1.28	*RPS6*	1.37	*EF-1α*	0.93	*AK*	0.92
	9	*18S*	1.39	*EF-1α*	1.49	*18S*	1.16	*18S*	1.02
	10	*28S*	1.45	*RPL13*	1.50	*28S*	1.24	*28S*	1.08
	11	*GST*	2.06	*GST*	2.13	*GST*	1.92	*GST*	1.26
Population	1	*AK*	1.26	*18S*	0.61	*AK*	0.33	*AK/*	0.58
	2	*TATA*	1.30	*RPS6*	0.65	*RPS18*	0.48	*TATA*	
	3	*RPS18*	1.31	*RPS18*	0.67	*TATA*	0.52	*RPS18*	0.72
	4	*EF-1α*	1.39	*TATA*	0.91	*EF-1α*	0.72	*EF-1α*	0.84
	5	*18S*	1.43	*AK*	0.94	*18S*	0.92	*18S*	1.02
	6	*GAPDH*	1.60	*28S*	0.95	*GAPDH*	1.22	*RPS6*	1.14
	7	*RPS6*	1.61	*RPL13*	0.98	*GST*	1.27	*RPL13*	1.17
	8	*GST*	1.64	*EF-1α*	1.06	*RPS6*	1.31	*GAPDH*	1.28
	9	*RPL13*	1.65	*GST*	1.53	*RPL13*	1.32	*GST*	1.33
	10	*28S*	2.06	*GAPDH*	1.54	*28S*	1.91	*28S*	1.44
	11	*β-actin*	2.27	*β-actin*	2.32	*β-actin*	2.15	*β-actin*	1.59
Tissue	1	*RPL13*	1.10	*28S*	0.37	*RPS18*	0.14	*RPS18/*	0.21
	2	*GAPDH*	1.12	*18S*	0.66	*RPL13*	0.14	*EF-1α*	
	3	*RPS18*	1.13	*GAPDH*	1.39	*GAPDH*	0.16	*RPL13*	0.28
	4	*β-actin*	1.17	*RPS18*	1.42	*β-actin*	0.18	*GAPDH*	0.35
	5	*EF-1α*	1.18	*β-actin*	1.42	*TATA*	0.38	*β-actin*	0.39
	6	*TATA*	1.20	*EF-1α*	1.45	*EF-1α*	0.40	*TATA*	0.41
	7	*RPS6*	1.28	*RPS6*	1.45	*RPS6*	0.62	*RPS6*	0.45
	8	*28S*	2.36	*RPL13*	1.50	*28S*	2.16	*GST*	0.86
	9	*GST*	2.44	*TATA*	1.50	*AK*	2.19	*AK*	1.14
	10	*AK*	2.44	*GST*	2.46	*GST*	2.23	*28S*	1.42
	11	*18S*	2.64	*AK*	2.47	*18S*	2.51	*18S*	1.64
Wing dimorphism	1	*GAPDH*	1.63	*28S*	0.47	*TATA*	0.15	*TATA/*	0.30
	2	*TATA*	1.66	*β-actin*	1.75	*GAPDH*	0.21	*GAPDH*	
	3	*RPS6*	1.70	*RPS18*	1.89	*RPS6*	0.34	*RPS18*	0.59
	4	*RPL13*	1.82	*TATA*	2.04	*RPL13*	0.58	*β-actin*	0.71
	5	*RPS18*	1.87	*RPS6*	2.11	*RPS18*	0.81	*RPS6*	0.81
	6	*β-actin*	1.93	*GAPDH*	2.16	*β-actin*	0.83	*RPL13*	0.89
	7	*EF-1α*	1.95	*18S*	2.43	*EF-1α*	1.18	*EF-1α*	0.99
	8	*AK*	2.40	*RPL13*	2.51	*AK*	1.88	*AK*	1.20
	9	*GST*	2.74	*EF-1α*	2.83	*28S*	2.16	*GST*	1.37
	10	*28S*	2.93	*AK*	3.31	*GST*	2.45	*28S*	1.66
	11	*18S*	5.68	*GST*	3.71	*18S*	5.59	*18S*	2.39
Antibiotic	1	*RPS6*	0.55	*28S*	0.27	*EF-1α*	0.04	*EF-1α/*	0.04
	2	*EF-1α*	0.56	*18S*	0.28	*RPS6*	0.08	*RPS18*	
	3	*RPS18*	0.57	*β-actin*	0.66	*RPL13*	0.12	*RPL13*	0.12
	4	*RPL13*	0.57	*AK*	0.67	*RPS18*	0.13	*RPS6*	0.15
	5	*GAPDH*	0.57	*RPS6*	0.74	*TATA*	0.14	*GAPDH*	0.17
	6	*TATA*	0.62	*GAPDH*	0.77	*GAPDH*	0.18	*TATA*	0.21
	7	*β-actin*	0.63	*TATA*	0.81	*β-actin*	0.23	*β-actin*	0.26
	8	*28S*	0.87	*RPL13*	0.85	*AK*	0.61	*28S*	0.38
	9	*AK*	0.89	*EF-1α*	0.87	*28S*	0.74	*18S*	0.46
	10	*18S*	0.94	*RPS18*	0.89	*18S*	0.84	*AK*	0.52
	11	*GST*	2.07	*GST*	2.05	*GST*	2.04	*GST*	0.80
Insecticide	1	*GAPDH*	1.12	*18S*	0.32	*GAPDH*	0.28	*GAPDH*	0.27
	2	*β-actin*	1.14	*β-actin*	0.73	*β-actin*	0.35	*/TATA*	
	3	*TATA*	1.14	*28S*	0.76	*TATA*	0.38	*β-actin*	0.45
	4	*28S*	1.22	*RPS18*	0.80	*28S*	0.56	*28S*	0.53
	5	*EF-1α*	1.29	*TATA*	0.99	*EF-1α*	0.62	*18S*	0.70
	6	*RPL13*	1.46	*GAPDH*	0.99	*RPL13*	0.96	*RPS18*	0.82
	7	*AK*	1.54	*EF-1α*	1.36	*AK*	1.09	*EF-1α*	0.94
	8	*18S*	1.57	*RPL13*	1.71	*18S*	1.20	*RPL13*	1.05
	9	*RPS18*	1.59	*AK*	1.84	*RPS18*	1.26	*AK*	1.13
	10	*RPS6*	2.13	*RPS6*	2.08	*RPS6*	1.91	*RPS6*	1.30
	11	*GST*	2.50	*GST*	2.46	*GST*	2.35	*GST*	1.52
Starvation	1	*GAPDH*	1.39	*28S*	0.49	*GAPDH*	0.11	*GAPDH*	0.22
	2	*TATA*	1.44	*18S*	0.64	*RPS18*	0.20	*/TATA*	
	3	*β-actin*	1.45	*GST*	0.93	*β-actin*	0.26	*β-actin*	0.44
	4	*RPS18*	1.48	*RPS18*	1.78	*TATA*	0.37	*RPS18*	0.52
	5	*EF-1α*	1.64	*β-actin*	1.83	*EF-1α*	0.87	*EF-1α*	0.73
	6	*RPS6*	1.94	*GAPDH*	2.25	*RPS6*	1.63	*RPS6*	1.00
	7	*RPL13*	2.04	*TATA*	2.30	*RPL13*	1.75	*RPL13*	1.12
	8	*AK*	2.25	*EF-1α*	2.48	*GST*	1.90	*AK*	1.22
	9	*GST*	2.29	*RPS6*	3.32	*AK*	2.07	*GST*	1.51
	10	*28S*	2.38	*RPL13*	3.34	*28S*	2.09	*28S*	1.72
	11	*18S*	2.81	*AK*	3.67	*18S*	2.69	*18S*	1.92
Temperature	1	*TATA*	1.28	*18S*	0.72	*TATA*	0.50	*TATA/*	0.92
	2	*RPS6*	1.37	*TATA*	0.80	*AK*	0.72	*RPS6*	
	3	*AK*	1.38	*RPS6*	0.85	*RPS6*	0.74	*AK*	1.00
	4	*GAPDH*	1.48	*28S*	0.88	*GAPDH*	0.97	*28S*	1.09
	5	*β-actin*	1.56	*RPS18*	0.97	*β-actin*	1.08	*18S*	1.14
	6	*28S*	1.57	*GAPDH*	1.08	*28S*	1.13	*GAPDH*	1.23
	7	*18S*	1.60	*AK*	1.10	*18S*	1.17	*RPS18*	1.30
	8	*RPL13*	1.69	*β-actin*	1.30	*RPL13*	1.28	*β-actin*	1.35
	9	*RPS18*	1.77	*RPL13*	1.48	*RPS18*	1.44	*RPL13*	1.43
	10	*GST*	1.91	*EF-1α*	1.63	*GST*	1.61	*GST*	1.52
	11	*EF-1α*	1.96	*GST*	1.88	*EF-1α*	1.65	*EF-1α*	1.60
All conditions	1	*TATA*	1.54	*28S*	0.71	*TATA*	0.39	*β-actin/*	0.78
	2	*GAPDH*	1.68	*18S*	0.73	*GAPDH*	0.86	*GAPDH*	
	3	*β-actin*	1.74	*RPS18*	1.58	*β-actin*	0.90	*TATA*	0.87
	4	*RPS18*	1.80	*TATA*	1.59	*RPS18*	1.04	*RPS18*	0.99
	5	*EF-1α*	1.84	*β-actin*	1.68	*EF-1α*	1.11	*EF-1α*	1.16
	6	*RPL13*	1.85	*RPS6*	1.84	*RPL13*	1.15	*RPL13*	1.27
	7	*RPS6*	1.96	*GAPDH*	1.87	*RPS6*	1.34	*RPS6*	1.35
	8	*AK*	2.08	*RPL13*	2.08	*AK*	1.50	*AK*	1.44
	9	*28S*	2.35	*EF-1α*	2.15	*28S*	1.87	*28S*	1.63
	10	*18S*	2.68	*AK*	2.46	*18S*	2.33	*18S*	1.82
	11	*GST*	3.17	*GST*	3.02	*GST*	2.89	*GST*	2.06

**TABLE 3:** Recommended reference genes for *R. padi* under various experimental conditions.

Conditions	Reference gene	Conditions	Reference gene
Developmental stages	*β-Actin*, *GAPDH*	Antibiotic	*EF-1α*, *RPS6*
Population	*AK*, *TATA*	Insecticide	*GAPDH*, *β-Actin*
Tissue	*RPS18*, *RPL13*	Starvation	*GAPDH*, *TATA*, *RPS18*
Wing dimorphism	*TATA*, *GAPDH*	Temperature	*TATA*, *RPS6*, *AK*
All conditions	*TATA*, *GAPDH*		

#### Population Analysis

On the basis of the geNorm, NormFinder, and ΔCt analyses, *AK*, *TATA*, and *RPS18* were the most stable genes (Table 2). However, the BestKeeper analysis indicated *18S* and *RPS6* were the most stable genes (Table 2). All four analyses revealed *β-actin* was the least stable gene (Table 2). The rank order for gene stability in the populations determined using RefFinder was as follows (most to least stable): *AK*, *TATA*, *RPS18*, *18S*, *EF-1α*, *RPS6*, *GAPDH*, *RPL13*, *GST*, *28S*, and *β-actin* (Figure 2B). The geNorm data indicated that the pairwise variation value for V2/3 was less than the proposed 0.15 cut-off, whereas the other values exceeded 0.15, except for V4/5 (Figure 3). The RefFinder analysis suggested that *AK* and *TATA* are required for the normalization of target gene expression levels in different populations (Table 3).

#### Tissue Analysis

Both ΔCt and NormFinder identified *RPL13*, *RPS18*, and *GAPDH* as the most stable genes across tissue samples (Table 2). The geNorm analysis also identified *RPL13* as a stable gene, but *RPL18* and *EF-1α* were more stable (Table 2). In contrast, BestKeeper revealed *28S* and *18S* were the most stable genes (Table 2). The rank order for gene stability among the examined tissues based on the RefFinder results was as follows (most to least stable): *RPS18*, *RPL13*, *GAPDH*, *EF-1α*, *β-actin*, *28S*, *TATA*, *RPS6*, *18S*, *GST*, and *AK* (Figure 2C). The geNorm analysis indicated that the pairwise value of V2/3 was less than the proposed 0.15 cut-off (Figure 3). The RefFinder analysis suggested *RPS18* and *RPL13* are required for normalizing target gene expression levels in various *R. padi* tissues (Table 3).

#### Wing Dimorphism Analysis

The ΔCt analyses identified *GAPDH* and *TATA* as the most stable genes and *18S* as the least stable gene. Similar results were obtained from geNorm and NormFinder (Table 2). In contrast, BestKeeper detected *28S* and *β-actin* as the most stable genes (Table 2). The RefFinder results for wing dimorphism indicated the rank order for gene stability was as follows (most to least stable): *TATA*, *GAPDH*, *RPS6*, *RPS18*, *β-actin*, *RPL13*, *28S*, *EF-1α*, *AK*, *GST*, and *18S* (Figure 2D). Similar to the tissue results, the geNorm data indicated that the pairwise value of V2/3 was less than the proposed 0.15 cut-off (Figure 3). On the basis of the RefFinder analysis, *TATA* and *GAPDH* are required for normalizing target gene expression levels between the wing and wingless samples (Table 3).

#### Antibiotic Treatment Analysis

All four analyses identified *GST* as the least stable gene (Table 2). Reference genes *EF-1α* and *RPS18* were identified as the most stable genes by geNorm (Table 2). In terms of gene stability, *RPS6* and *EF-1α* were ranked number 1 and 2, respectively, according to the ΔCt method. These two genes were also the most stable based on the NormFinder results, although the positions were flipped (Table 2). The BestKeeper analysis identified *28S* and *18S* as the most stable genes. The RefFinder analysis of the antibiotic treatments indicated the rank order for gene stability was as follows (most to least stable): *EF-1α*, *RPS6*, *RPS18*, *RPL13*, *28S*, *GAPDH*, *β-actin*, *TATA*, *18S*, *AK*, and *GST* (Figure 2E). The geNorm analysis revealed that all pairwise variation values were less than the proposed 0.15 cut-off, except for V10/11 (Figure 3). Furthermore, the RefFinder results suggested that *EF-1α* and *RPS6* are required for the normalization of gene expression across antibiotic treatments (Table 3).

#### Insecticide Treatment Analysis

The stability rankings determined by the four analyses identified *GST* as the most unstable gene. With the exception of BestKeeper, the analyses suggested *GAPDH* and *TATA* were the most stable genes (Table 2). The second most stable gene was *β-actin*. Notably, BestKeeper revealed *18S* as the most stable gene, whereas this gene was only moderately stable according to the other analyses (Table 2). On the basis of the RefFinder data, the rank order for gene stability among insecticide treatments was as follows (most to least stable): *GAPDH*, *β-actin*, *TATA*, *28S*, *18S*, *EF-1α*, *RPS18*, *RPL13*, *AK*, *RPS6*, and *GST* (Figure 2F). The geNorm analysis indicated that the pairwise variation value of V2/3 was 0.15, which was the proposed cut-off (Figure 3). The RefFinder results implied *GAPDH* and *β-actin* are required for normalizing target gene expression levels following insecticide treatments (Table 3).

#### Starvation Analysis

Regarding the starvation effects, the ΔCt, geNorm, and NormFinder analyses indicated that the most stable genes were *GAPDH*, *TATA*, *β-actin*, and *RPS18*, whereas the least stable gene was *18S* (Table 2). However, *18S* was the second most stable gene in the rankings determined using BestKeeper, with only *28S* more stable (Table 2). The rank order for gene stability under starvation conditions determined using RefFinder was as follows (most to least stable): *GAPDH*, *TATA*, *RPS18*, *β-actin*, *EF-1α*, *28S*, *RPS6*, *GST*, *18S*, *RPL13*, and *AK* (Figure 2G). The geNorm analysis revealed that the pairwise variation value for V3/4 was less than the proposed 0.15 cut-off (Figure 3). The RefFinder analysis indicated that target gene expression levels under starvation stress conditions should be normalized against the expression of *GAPDH*, *TATA*, and *RPS18* (Table 3).

#### Temperature Analysis

All analyses except for BestKeeper indicated that *TATA*, *RPS6*, and *AK* were the most stable genes. BestKeeper replaced *AK* with *28S* among the most stable genes (Table 2). The least stable gene was *EF-1α* according to ΔCt, NormFinder, and geNorm. In contrast, BestKeeper identified *GST* as the least stable gene (Table 2). The RefFinder data indicated the rank order for gene stability among temperatures was as follows (most to least stable): *TATA*, *RPS6*, *AK*, *18S*, *GAPDH*, *28S*, *β-actin*, *RPS18*, *RPL13*, *GST*, and *EF-1α* (Figure 2H). The geNorm analysis revealed that the pairwise variation value of V4/5 was less than the proposed 0.15 cut-off (Figure 3). The RefFinder analysis suggested that *TATA*, *RPS6*, and *AK* are required for normalizing target gene expression levels in temperature-treated *R. padi* (Table 3).

#### Overall Ranking of *R. padi* Reference Genes

On the basis of the RefFinder analysis, the overall rank order for the stability of *R. padi* reference genes was as follows (most to least stable): *TATA*, *GAPDH*, *β-actin*, *RPS18*, *28S*, *EF-1α*, *RPL13*, *18S*, *RPS6*, *AK*, and *GST* (Figure 2I). The geNorm analysis indicated that all pairwise variation values were less than the proposed 0.15 cut-off (Figure 3). The RefFinder data suggested that *TATA* and *GAPDH* are suitable internal reference genes for normalizing target gene expression levels in *R. padi* (Table 3).

## Discussion

Quantitative real-time PCR is one of the most important and reliable techniques for quantifying the expression of a target gene under different experimental conditions. However, obtaining a robust and reliable estimate for gene expression levels under different conditions requires a data normalization using an appropriate reference gene. Therefore, identifying suitable housekeeping genes is critical for qRT-PCR analyses. Because housekeeping genes are constitutively expressed to maintain basic cellular activities, they have traditionally been used as internal reference controls (Vandesompele et al., 2002b; Fu et al., 2013; Yang et al., 2014). There are several reports describing the application of qRT-PCR assays to clarify the expression of genes associated with diverse biological processes (Ross et al., 2000; Solanas et al., 2001; Bustin et al., 2009; Mao and Zeng, 2014). Reference genes used for molecular investigations can influence the accuracy of target gene expression levels (Bustin, 2002; Vandesompele et al., 2002a, b; Gutierrez et al., 2008). Hence, a stable reference gene is an important prerequisite for gene expression investigations. Moreover, all stable reference genes used for normalizing gene expression data should be evaluated for each experimental condition.

To date, reliable reference genes have been identified for many Hemiptera species, including *Metopolophium dirhodum* (Li et al., 2020), *M. persicae* (Kang et al., 2017), *Aphis gossypii* (Ma et al., 2016), *Acyrthosiphon pisum* (Yang et al., 2014), *Aphis glycines* (Bansal et al., 2012), *Megoura viciae* (Cristiano et al., 2016), *Bemisia tabaci* (Li et al., 2013), *Nilaparvata lugens* (Yuan et al., 2014), *Sogatella furcifera* (An et al., 2015), and *Diaphorina citri* (Bassan et al., 2017). *R. padi* is one of the most important wheat pests. Thus, in-depth investigations of *R. padi* at the molecular level may generate information relevant for improving pest control treatments. Accordingly, the stability of reference gene expression levels in *R. padi* under diverse experimental conditions should be evaluated. In a previous study, the expression stability of only four housekeeping genes was assessed for viruliferous winged and wingless *R. padi* adults (Wu et al., 2014). In the current study, we examined the stability of 11 candidate reference gene expression levels in *R. padi* in response to various conditions.

The *TATA* gene is required for initiating the RNA polymerase (I, II, and III)-mediated transcription of genes with promoters with or without a TATA box (Pugh, 2000; Hochheimer and Tjian, 2003). In this study, we determined that *TATA* expression was highly stable in all conditions (Figure 2I), which is consistent with the results of earlier investigations. For example, *TATA* was identified as a stable reference gene for normalizing target gene expression in *D. melanogaster* treated with acetic acid (Kim et al., 2020). Additionally, *TATA* expression was revealed to be moderately stable in female and male *P. solenopsis* at different developmental stages (Zheng et al., 2019). Regarding *P. tsushimanus*, *TATA* is the most stable reference gene for analyzing gene expression among plant samples, but it is the least stable reference gene for gene expression analyses of developmental stages (Chen C. et al., 2020). Thus, the stability of reference gene expression must be evaluated for specific experimental conditions.

The *GAPDH* gene was identified as a stably expressed reference gene across most sample sets in the current study (Figure 2). This is consistent with the common usage of *GAPDH* as an ideal reference gene across a range of experimental treatments and conditions (Price and Wilson, 2014). Additionally, *GAPDH* activity is vital for membrane development, microtubule processing, and reactions catalyzed by phosphotransferases and kinases (Colell et al., 2009). Several recent studies have used *GAPDH* as a reference control for gene expression analyses (Han et al., 2020; Pinheiro and Siegfried, 2020; Yin et al., 2020). In *Lucilia cuprina*, *GAPDH* expression is highly variable, making the gene a poor choice as a reference control (Bagnall and Kotze, 2010). Moreover, the *GAPDH* gene is reportedly not stably expressed in temperature-stressed *Galeruca daurica* (Tan et al., 2016).

In the current study, *β-actin*, *EF-1α*, and *AK* expression levels were moderately stable (Figure 2). These genes have been frequently selected as reference genes for many other insect species. The *β-actin* gene encodes a major component of the protein scaffolding that supports cells and determines their shape (Zhu et al., 2014). We identified the *β-actin* gene as the most stable housekeeping gene only during examinations of different *R. padi* developmental stages (Figure 2A). The protein encoded by the *EF-1α* gene belongs to the GTP-binding elongation factor family and is localized in the cytoplasm, where it functions as an essential enzyme during the elongation phase of protein synthesis. The results of our analysis of *EF-1α* expression in *R. padi* were similar to the findings of an earlier study (Wu et al., 2014), in which *EF-1α* was identified as one of the most stable reference genes in viruliferous *R. padi*, but it was not appropriate for qRT-PCR assays. In contrast, we determined that *EF-1α* is a suitable reference gene for analyzing the effects of antibiotic treatments on *R. padi* (Figure 2E). Additionally, the *AK* gene encodes a phosphagen kinase in invertebrates, and it has rarely been used as a reference gene (Li et al., 2020). In the *Bombus terrestris* labial gland and fat body, *AK* was observed to be the most stably expressed gene (Hornakova et al., 2010). In our study, *AK* was detected as the most stable gene during analyses of aphid populations (Figure 2B). These results imply that reference genes should be used only for specific experimental conditions.

Ribosomal proteins are among the most highly conserved proteins across all life forms (Yuan et al., 2014; Shi et al., 2016). An earlier study revealed that ribosomal protein-encoding genes have been the most stably expressed and widely used reference genes for molecular studies of insects during the past 10 years (Vilcinskas et al., 2013; Yuan et al., 2014; Lu et al., 2018; Qu et al., 2018). Consistent with these earlier findings, we identified *RPS18* and *RPL13* as the most stable reference genes in *R. padi* across various tissues (Figure 2C). Moreover, *RPS6* was detected as the second most stable gene in response to antibiotic and temperature treatments (Figures 2E,H), but it was among the least stable genes during analyses of *R. padi* developmental stages (Figure 2A) and the effects of insecticide treatments (Figure 2F). Interestingly, an earlier investigation of the honey bee (Moon et al., 2018) suggested *RPS18* and/or *GAPDH* are useful reference genes for analyzing gene expression in the whole body, which is consistent with our results for different aphid body parts (Figure 2C).

The *18S* and *28S* sequences encode ribosomal RNAs that contribute to protein synthesis. These sequences are highly expressed in all biological cells. Although ribosomal RNAs are generally considered to be reliable internal controls, several studies have shown that the commonly used reference genes may not be applicable to different experimental conditions. Our analyses demonstrated that *18S* and *28S* had the highest relative expression levels among experiments (Figure 1), with the lowest Ct values (8.08 and 9.08, respectively), which precludes their use as appropriate reference genes in *R. padi* qRT-PCR assays. This result is consistent with the findings of a previous study on developing long bones in rats under physiological conditions and following prenatal dexamethasone exposures (Han et al., 2020). It is also in accordance with the data produced during an examination of the rice moth *Corcyra cephalonica* (Vantaku et al., 2019). Furthermore, *28S* was detected as one of the two least suitable reference genes in *A. gossypii* under most experimental conditions (Ma et al., 2016). A previous study also confirmed that *18S* cannot serve as a reference gene in *R. padi* (Wu et al., 2014). However, both *18S* and *28S* are reportedly stably expressed in *H. axyridis* feeding on different diets (Liang et al., 2019).

The *GST* gene encodes a major detoxifying enzyme in most organisms, including plants and animals (Balakrishnan et al., 2018). In our study, *GST* was detected as an inappropriate reference gene (Figure 2), but it may be suitable in other insects and/or under different conditions. In a previous study on *B. tabaci*, *GST* was identified as the least stably expressed gene during analyses of various hosts, but it was the most stably expressed gene across all samples (Kaur et al., 2019).

Our results demonstrate that the rank order of reference genes may change depending on the biological samples and experimental conditions. The rank order may also differ among the methods used for analyses. For example, the ΔCt method detected *GAPDH* and *TATA* as the most and second most stably expressed genes, which was in contrast to the NormFinder results. The geNorm analysis indicated these two genes were the most stable during the examination of wing dimorphism (Table 2). However, *28S* was rated as the best reference gene by BestKeeper (Table 2). Ultimately, RefFinder revealed the gene stability rank order was as follows (most to least stable): *TATA*, *GAPDH*, *RPS6*, *RPS18*, *β-actin*, *RPL13*, *28S*, *EF-1α*, *AK*, *GST*, and *18S* (Figure 2D). The differences among the programs and methods used for ranking genes are largely the result of the diversity in the statistical algorithms. To analyze the stability of reference genes, BestKeeper analyzes the reference genes individually. The ΔCt method, NormFinder, and geNorm compare the pairwise variation between two reference genes. RefFinder conducts comprehensive evaluations by combining the above four results.

The MIQE guidelines state that normalization using a single reference gene is unreliable (Bustin et al., 2009). Previous studies proved that either too few or too many reference genes may influence the accuracy and reliability of data normalizations (Ling and Salvaterra, 2011; Fu et al., 2013). In recent years, the use of multiple reference genes instead of a single reference gene has become more common. Applying multiple reference genes minimizes biased normalizations and increases the reliability of qRT-PCR data under different conditions (Chang et al., 2017; Kang et al., 2017; Wang G. et al., 2020). The optimal number of reference genes under specific experimental conditions can be determined using the geNorm algorithm, which calculates the pairwise variation Vn/n+1 based on the normalization factors NFn and NFn + 1, with *n* ≥ 2. If Vn/n + 1 is less than 0.15, *n* is the optimal number of reference genes.

In this study, we identified internal reference genes that are suitable for normalizing and quantifying gene expression levels in *R. padi* (Table 3). These findings suggest that many candidate genes should not be used as default reference genes because their expression is highly variable under certain conditions. Additionally, there is no universal reference gene that is stably expressed in all conditions or in all organisms. Therefore, putative reference genes must be validated before each qRT-PCR analysis to ensure they are appropriate for the experimental conditions. Another important consideration for qRT-PCR studies is the optimal number of reference genes.

## Data Availability Statement

The original contributions presented in the study are included in the article/supplementary material, further inquiries can be directed to the corresponding author/s.

## Author Contributions

XZ and FY conceived and designed the research. ML, XrL, CW, QL, and SZ conducted the experiments. ML and XL analyzed the data. ML wrote the manuscript. YZ, XL, FY, and XZ revised the manuscript. All authors have read and approved the manuscript.

## Conflict of Interest

The authors declare that the research was conducted in the absence of any commercial or financial relationships that could be construed as a potential conflict of interest.

## Footnotes

1 https://www.heartcure.com.au/for-researchers
